# Following patients with acute abdominal pain in emergency departments

**DOI:** 10.1186/1757-7241-20-S2-P23

**Published:** 2012-04-16

**Authors:** Helen Schultz, Birthe D Pedersen, Niels Qvist, Christian Backer Mogensen

**Affiliations:** 1 Enheden for Sygeplejeforskning, Klinisk Institut, Syddansk Universitet, Campusvej 55, 5230 Odense C, Denmark

## Background

The Danish health care system is reorganizing care and treatment of acute patients in the hospitals by replacing the emergency departments and departments that receive acute patients with emergency departments with observation units.

The objective is to study the professional efforts and experiences of patients with acute abdominal pain in an acute visitation unit for abdominal surgery (AVUAS) versus an emergency department with an observation unit (ED).

## Methods

Research of the acute process is supported by field observations with focus on content, duration and time concerning patient contact with professionals. Twenty patients are included. The same twenty patient’s experience are studied by two interviews. First interview takes place at discharge, where the focus is the patient’s experience of the acute process. The second interview takes place three months after discharge, with focus on the time after the discharge.

The professional effort of the acute patients is researched by 160 medical records, where care and treatment, length of stay and re-admissions are registered.

## Results

The analysis of data from the field study and the interviews are still in progress, and the analysis of medical records has not started yet.

Preliminary finding shows that the intervening period to a specialist in ED was 136 minutes (95% CI: 64-208 minutes) and at AVUAS 118 minutes (95% CI: 73-163 minutes) - A non-significant difference. In ED the patients were seen by a physician before seen by a specialist. Nurses spent about 32 minutes with patients at admission in ED and about 15 minutes in AVUAS - A non-significant difference.

In ED patient experience is prompt examination, but they need to repeat themselves – In AVUAS patient experience is much waiting time.

## Conclusion

Preliminary conclusions indicate that at admission the patients in ED have to wait less time to be seen by a physician, they have to wait longer time to be seen by a specialist and they spent more time with the nurses. At admission, patients in ED experience prompt and a bit overwhelming examination. In AVUAS patients experience a long waiting time.

